# Food hypersensitivity in patients treated with specific allergen immunotherapy (SIT) for aeroallergens

**DOI:** 10.1186/2045-7022-3-S3-P64

**Published:** 2013-07-25

**Authors:** J Gocki, Z Bartuzi

**Affiliations:** 1 Allergy, Clinical Immunology and Internal Diseases, Collegium Medicum. University Nikolaus Copernicus, Bydgoszcz, Poland

## Background

Coexistence of food hypersensitivity are frequently observed in patients with allergy to plant and animals. Principal method of cure of aeroallergens hypersensitivity is specific allergy immunotherapy (SIT). Effectiveness of this therapy is assessed based on clinical symptoms abated. Sometimes we observed less effect this method of treatment in some patients, probably connected with coexistence of food hypersensitivity, which could be cross allergy or co-allergy.

## Aim

The aim of this study was assessment prevalence of food hypersensitivity in patients with SIT.

## Methods

We analysis case history 55 patients with SIT for aeroallergens, in age 18 to 72 years (medium 55 years), 28 women in age 18 to 62 years (medium 36 years), and 27 man in age 18 to 72 years (medium 34 years).

## Results

Inclusion criteria to SIT in 41 patients was allergic rhinitis (AR), in 12 patients – coexistence of asthma and AR, and in 2 patients – asthma. More frequently we observed allergy for several aeroallergens – in 32 patients. Only in 23 patients have allergy for one aeroallergen. Most of patients was treated SIT for grass allergy -16 patients, dust mites – 11, trees – 10, trees and grasses – 6, ragweed, cat – 4, and for one to: grasses and ragweed, trees and ragweed, alternaria. Prevalence of food hypersensitivity in analyzing group of patients treated with SIT for aeroallergens was 27,2% (15 patients). More frequently we observed food hypersensitivity in patients with plant allergy – 33,3% (13 patients). Between patients treated with SIT due to house dust mites allergy, we observed food hypersensitivity in 2 patients (18,1%), and we didn`t observed coexistence of food hypersensitivity in patients treated with SIT for cat allergy and alternaria.

## Conclusion

Prevalence of food hypersensitivity in patients treated with SIT for aeroallergens was 27,2%. More frequently it was observed in patients with plant allergy – 33,3%. Coexistence food hypersensitivity in this group of patients could influence on less effective results of SIT, because probably food hypersensitivity is important element supporting of allergy process in this patients. This idea is strong argument for intensive cure of food hypersensitivity in this patients.

## Disclosure of interest

None declared.

